# Three-Dimensional Bioprinting in Medicine: A Comprehensive Overview of Current Progress and Challenges Faced

**DOI:** 10.7759/cureus.41624

**Published:** 2023-07-10

**Authors:** Rithik S Veeravalli, Bhuvanasai Vejandla, Sarah Savani, Aditya Nelluri, Nikhil Chowdary Peddi

**Affiliations:** 1 Medicine, University of Cincinnati College of Medicine, Cincinnati, USA; 2 Emergency, Loyola University Chicago, Chicago, USA; 3 Medicine, Loyola University Chicago, Chicago, USA; 4 Medicine, Sri Siddhartha medical college, Tumakuru, IND; 5 Pediatrics, St. Barnabas Hospital Health System, New York, USA

**Keywords:** bio engineering, bioinks, tissue engineering & regenerative medicine, regenerative medicine treatments, 3d-bioprinting

## Abstract

The shortage of organs for transplantation is a global crisis, with an increasing demand and an inadequate supply of organ donors. The convergence of biology and engineering has led to the emergence of 3D bioprinting, which enables the precise and customizable construction of biological structures. Various 3D bioprinting techniques include inkjet printing, extrusion printing, and laser-assisted bioprinting (LAB). Although it has the potential for many benefits, 3D bioprinting comes with its own set of challenges and requirements, specifically associated with the bioprinting of various tissues. The challenges of bioprinting include issues with cells, bioinks, and bioprinters, as well as ethical concerns, clinical efficacy, and cost-effectiveness, making it difficult to integrate 3D bioprinting into widespread clinical practice. Three-dimensional bioprinting holds great promise in addressing the organ shortage crisis, and its applications extend beyond organ transplantation to include drug screening, disease modeling, and regenerative medicine. However, further research is needed to overcome the technical, biological, and ethical challenges associated with 3D bioprinting, paving the way for its widespread clinical implementation. This article discusses the processes and challenges of bioprinting as well as the current research direction in the field.

## Introduction and background

The shortage of organs for transplantation is a global crisis with a rapidly increasing demand and an inadequate supply of organ donors. In the United States, less than one-third of patients on the waiting list receive transplanted organs, whereas only one out of every 104 people suffering from end-stage organ failure receives an organ transplant in China [1]. According to statistics, 74.63% of organ donation candidates failed to receive a transplant in the United States in 2015 and 19.89% in the UK in 2018 [2]. As of October 2022, the national organ transplant waitlist in the United States exceeded 110,000 people. Tragically, despite being on the waitlist, approximately 20 individuals lost their lives each day. It has been reported that the number of people waiting for organ transplants has increased by 7% over the last ten years, but that the number of donors and transplants has remained relatively the same [3].

Biological tissue can now be created through the convergence of biology and engineering thanks to bioprinting, regenerative medicine, and materials science. However, translating 3D-printed constructs into clinical practice presents several challenges [4].

Tissue engineering methods have demonstrated the potential for creating artificial organs. The fundamental idea behind traditional tissue engineering techniques involves placing living cells and/or biologically active substances onto a porous scaffold, which helps repair and regenerate damaged tissues. There are certain limitations associated with traditional methods, such as inconsistent cell distribution, low cell density, and challenges in integrating vascular and neural networks [5].

To address the challenges of traditional tissue engineering methods, 3D bioprinting, a type of additive technique, has revolutionized the field by enabling the precise and customizable construction of biological structures. Bioprinted models mimic 3D microenvironments for an accurate representation of human physiology, surpassing 2D cell cultures and animal models [6].

Stereolithography, one of the earlier forms of additive manufacturing, was first invented in 1983 [7]. It operates by utilizing a liquid resin photocuring process in which a reservoir holds the liquid resin (a blend designed for polymerization or cross-linking). To initiate photopolymerization, a laser is systematically moved across the surface of the resin, following a predetermined pattern. After its invention, fused deposition modeling (FDM) - also known as 3D printing - was patented in 1989 and developed for modeling and prototyping in order to produce complex geometrical, low-cost, and easy-to-operate parts for applications in the aerospace, medical, and automotive industries [6]. Laser-based bioprinting emerged in the 1990s as a method for fabricating cells [8]. Three-dimensional bioprinting modalities can be classified as laser-assisted bioprinting (LAB), inkjet bioprinting/droplet bioprinting, and extrusion-based bioprinting. LAB utilizes laser-induced forward transfer (LIFT) technology for direct writing and helps create customized 3D models. It involves three main components: a pulsed laser source, a ribbon, and a receiving substrate. By pulsing the laser beam onto the ribbon, the metal layer on the hydrogel is vaporized, generating a high-pressure bubble that ejects bioink droplets onto the receiving substrate [9]. Inkjet bioprinting, introduced in the early 2000s, is a widely used droplet-based printing system. It employs noncontact reprographic techniques to reproduce patterns from digital data and construct structures layer by layer. Inkjet bioprinting imposes minimal requirements on the printing surface as a noncontact technology. By manipulating bioink properties and printing patterns, it can readily create gradients within a 3D structure [10]. Lastly, the extrusion-based bioprinting technique merges a fluid-dispensing system with an automated robotic system for extrusion and bioprinting. In the bioprinting process, a computer-controlled deposition system dispenses bioink, ensuring the accurate placement of cells within custom-shaped 3D structures in the form of cylindrical filaments [11].

Additionally, multiple materials can be printed simultaneously or sequentially using multi-head deposition systems (MHDSs). A custom-made bioprinting system is also available. The bioprinted construct is designed using a computer-aided design/computer-aided manufacturing (CAD/CAM) system [12]. Three-dimensional bioprinting enables precise layer-by-layer implantation of biological elements, biochemicals, and living cells for the creation of functional 3D structures. Key strategies include autonomous self-assembly, microtissue building blocks, and biomimicry [13].

The main component in 3D bioprinting, bioinks, prepared using hydrogels from natural or synthetic sources, play a crucial role in determining the printability and final properties of the printed constructs. It is a composite made up of biomaterials, cells, and other required components [14]. Although the choice of bioink and bioprinting process will vary greatly depending on the application, general features such as material properties, biological interaction, gelation, and viscosity are always important to consider.

Bioprinting, besides solving organ shortages, is useful for drug screening by tissue fabrication for drug testing, high-throughput assays, disease modeling, surgical training purposes, and cancer research as it can replicate the human microenvironment in vitro [15].

Furthermore, the field of bioprinting is advancing towards four-dimensional (4D) and five-dimensional (5D) printing, which allows for the creation of self-assembling constructs and controlled drug delivery systems. These advancements have the potential to revolutionize tissue engineering, regenerative medicine, and in vitro disease modeling [16].

An overview of 3D bioprinting is provided in this review, including ink-jet printing, extrusion printing, stereolithography, and laser-assisted bioprinting. It discusses the bioprinting of various tissues, highlighting the technological requirements and challenges involved. The review also addresses the current research direction in the field. By presenting the requirements and procedures, it aims to provide a broader perspective on 3D bioprinting.

## Review

Process of bioprinting

Three steps - pre-bioprinting, bioprinting, and post-bioprinting - can be used to denote the full bioprinting procedure.

Pre-bioprinting

Using the software, a digital design is created in the initial stage that will eventually be converted into a 3D structure by bioprinting. A biological model is obtained to create a design through a biopsy of the target tissue using computed tomography (CT) or magnetic resonance imaging (MRI) scans. These technologies offer the ability to transform photos into 2D formats, which are subsequently utilized to create model designs. Cells are then chosen and maintained in a suitable medium to produce bioink.

Bioprinting

The second phase involves loading bioink into the bioprinter, which then creates a 3D structure on a scaffold using the software's 2D design. The formation of different cell types in accordance with the target tissues and organs makes this a challenging stage.

Post-bioprinting

The bioprinted structure is stabilized in the final stage to preserve its shape and biological functions. Physical and chemical stimuli that permit cell rearrangement and maintain tissue growth are employed to stabilize the mechanical properties of the bioprinted structure [13].

There are three prominent techniques of bioprinting: laser-based bioprinting or laser-induced forward transfer, inkjet bioprinting/droplet-based bioprinting, and robotic dispensing/extrusion/deposition bioprinting [17].

Laser-induced forward transfer bioprinting employs a laser beam that undergoes focused transmission via a lens and is subsequently directed toward the lower surface of a transparent quartz substrate known as the donor slide. The donor slide is coated with bioink, which is a composite of live cells and either natural or synthetic polymers, such as hydrogels and matrigels. During the printing process, precise focalization of the laser beams onto the underside of the donor slide is facilitated by an f-theta lens. To optimize energy absorption and mitigate direct contact between the pulsed laser and the bioink, an energy-absorbing layer (EAL) can be interposed between the donor slide and the bioink. As the printing process is initiated, laser irradiation is targeted at the donor slide glass, with particular emphasis on the interface between the glass and the EAL. Upon the absorption of adequate laser energy by the bioink, the formation of a bubble occurs within the bioink layer. The ensuing expansion and subsequent collapse of the laser-induced bubble give rise to a transient jet flow. The material transfer process culminates when the jet reaches the underlying substrate, effectively depositing the bioink onto the receiving substrate [18].

Inkjet printing represents the second technique employed in bioprinting, characterized by the ejection of droplets containing cell suspension or bioink onto a substrate, hence referred to as drop-on-demand systems. Inkjet printing can be classified into two categories: thermal and piezoelectric inkjet printing, based on the mechanism utilized for droplet formation. In thermal inkjet printing, a vapor bubble is generated within the nozzle by a heater or heating filament, inducing the expulsion of the droplet from the nozzle and its subsequent deposition onto the substrate. Conversely, in piezoelectric inkjet printing, the application of electric pulses causes volume fluctuations in the piezoelectric crystal, resulting in the forceful ejection of the droplet through the nozzle [19].

Next in line are the robotic dispensing systems, which play a pivotal role in bioprinting. This particular technique involves the controlled extrusion of bioink from the nozzle through the application of mechanical force. The mechanical force employed can vary, encompassing pneumatic, piston-based, or screw-type mechanisms, each serving its purpose in facilitating precise and controlled bioink deposition. Pneumatic-driven extrusion employs compressed air to facilitate the dispensing of liquids. Typically, this system consists of a syringe filled with bioink, which is connected to an air pump via adapters and tubes. Pneumatic-driven systems work well with hydrogels that have shear-thinning characteristics because they continue to have a filamentous structure after extrusion. In the piston-driven extrusion system, a piston is connected to a motor through a guide screw. When the motor is activated, the rotational movement of the guide screw is converted into the linear motion of the piston, which in turn pushes the bioink out of the nozzle, resulting in filament formation. In addition to mechanically driven liquid dispensing systems, screw-driven devices provide improved volumetric control and make it easier to extrude biomaterials with high viscosities. The screw-driven system works on the same fundamentals as the piston-driven system, with the primary difference being that a screw, directly coupled to the motor, is used for extrusion instead of a piston [20].

Bioprinting material requirements

Bioink is at the core of the bioprinting process and is the main component needed for the target tissues and organs to be made. Essentially, bioink refers to the substance that is systematically deposited in a layer-by-layer manner throughout the bioprinting procedure. Bioink is typically composed of living cellular components, growth-supporting substances, and a reinforcing framework such as a hydrogel [21].

The hydrogel design can be composed of either naturally occurring or artificially made biomaterials [3]. The efficacy of natural biomaterials stems from their bioactivity. These materials bear a resemblance to the fluids encompassing cells and tissues in the body, thereby resulting in elevated biocompatibility. Nonetheless, they do exhibit mechanical fragility in comparison to their synthetic counterparts, which inhibits the stability of the printed construct. Thus, choosing which bioink to use is predicated on many factors [3]. An optimal bioink should possess specific physicochemical attributes, encompassing suitable mechanical, rheological, chemical, and biological properties. These characteristics should facilitate the generation of tissue constructs that exhibit adequate mechanical strength and durability while preserving the native mechanics of the target tissue, preferably with adjustable tunability. Additionally, the bioink should offer controllable gelation and stabilization properties to support the bioprinting process, enabling the creation of structures with high precision and fidelity. It is crucial for the bioink to demonstrate biocompatibility and, if required, biodegradability by replicating the natural microenvironment of the tissues. Furthermore, the bioink should be amenable to chemical modifications to meet the specific requirements of different tissue types. Lastly, the bioink should possess the potential for large-scale production, minimizing batch-to-batch variations [12].

Furthermore, the properties of the target tissue also play a large role in determining what material is required. Hard tissue bioinks, such as bone bioinks, are based on hydrogels, which commonly comprise a printable polymeric framework derived from gelatin methacrylate (gelMA), hyaluronic acid, or alginate units. These polymeric backbones are often enriched with bone-derived and/or osteogenic minerals and factors to enhance their functionality. Examples of such additives include beta-tricalcium phosphate (β-TCP), transforming growth factor-β (TGF-β), and bone morphogenetic proteins (BMPs) [22]. Conversely, soft tissue bioinks exhibit low shear stress levels even under moderate pressure, contributing to their desirable printing fidelity. These are frequently employed due to their ability to maintain excellent printing precision and support the viability of cells both in vitro and in vivo [22].

Challenges faced

There are many challenges that come across during the process of bioprinting. Cells, bioinks, and bioprinters are all necessary components of the bioprinting process, and each one poses biological, technical, and ethical issues as well as ambiguity regarding clinical efficacy and cost-effectiveness. As a result, there will be several difficulties in integrating 3D bioprinting into widespread clinical practice [4]. Additionally, the origins of both cell sources and bionic materials may lead to additional discussion within the healthcare environment [6]. When it comes to cell sourcing, the preferred cell type is from animal sources, as they are likely to enable greater mass production. The challenge that comes with the use of animal sources is the risk of disease transmission. Human sources, on the other hand, provide greater biocompatibility and the possibility for customization, but their usage is likely to be laden with stricter regulation, longer production periods, and higher prices. As discussed earlier, another challenge that comes with the process of bioprinting is the sourcing of bioinks. The first and most important factor is that the materials chosen for bioink synthesis must be biocompatible with the rest of the process. Many elements have gone through experimentation; the most common of these elements come from non-human species, such as gelatin from pig flesh and alginate from seaweed. These components' alien origins increase the risk of infection, inflammation, and immunogenicity [3]. Based on the role they play, bioinks can be classified into four categories. (1) Structural bioinks maintain the mechanical integrity of the construct and support cell adhesion, proliferation, and differentiation. They also mimic the extracellular matrix (ECM) during cell multiplication. (2) Temporary components that can be quickly removed to create interior gaps or channels in a 3D-printed design are known as fugitive bioinks or sacrificial bioinks. (3) Support bioinks are non-biologic substances with sufficient mechanical strength to withstand loads and offer mechanical support for delicate or intricate structures when printing. (4) Sacrificial and support bioinks are technically biomaterial inks rather than bioinks; functional bioinks deliver mechanical, biochemical, and electrical signals to affect cellular behavior post-printing. The rheological, mechanical, and biological characteristics of the bioink determine how functional the final printed tissues and organs are [4]. Currently, it is difficult for bioinks to satisfy all of these criteria, and an ideal bioink has not been identified [6].

The next challenge of Bioprinting lies in its integration into humans. The integration of vascular and neural networks in 3D-printed structures is the biggest obstacle [6]. Compared to other artificial implants, including stents and pacemakers, the biological component of implants makes integration and interactions more unpredictable when implanted into hosts. Processes including cell motility, post-printing phenotype, carcinogenic potential, and dysregulated differentiation will be impacted by variations in a patient's genotype. In the end, since bioprinting is a vaguely new concept, much research needs to be done before it can be a common implant procedure [3].

The bigger picture challenge is the ethical challenge. It starts off with the hesitancy of conducting clinical trials. The design of clinical trials will be difficult because it would be unethical to test tissue-engineered organ transplantation on healthy volunteers. In addition, because patient-specific cell populations would need to be used as the control, determining how much of the benefit is related to the patient's natural response to treatment and how much is due to the bioprinter product itself could be particularly challenging when assessing positive results from clinical trial patients [3]. Even if a plausible design for clinical trials is created, the inability of patients to withdraw after implantation and the difficulty in obtaining agreement for trial inclusion when the severity of problems is unknown are additional difficulties in organizing clinical trials [3]. Without proper clinical trials being conducted, there would be severe hesitancy for FDA regulation. The hesitancy for regulation will be doubled because of the constantly growing research and inventions in the field of bioprinting organs [4].

Future prospects of bioprinting

Many advancements have been made to address the effectiveness of the techniques within bioprinting and the functionality of the tissues and organs constructed. A downside of using synthetic or natural scaffolds in bioprinting is that the structural integrity is often too weak or not complex enough to sustain larger constructs. The integration of newer 3D bioprinting ideas with traditional techniques for scaffold fabrication offers a promising and innovative avenue for progress [23]. The relatively limited resolution of bioprinting poses challenges in directly fabricating sub-micrometer structures that accurately mimic the intricate characteristics of the natural extracellular matrix. This constraint may have a detrimental impact on cell adhesion and tissue regeneration. Conversely, established fabrication methods such as solvent casting, particulate leaching, gas foaming, electrospinning, phase separation, and freeze-drying offer greater flexibility in terms of creating diverse surface features. However, they fall short of providing precise control over scaffold pore size, geometry, and interconnectivity [23]. Combining the two methods, however, presents an avenue in which the integrity and effectiveness of the construct are both maximized.

Moreover, combining different fabrication processes within bioprinting can be employed to enhance both the practicability and effectiveness of the manufacturing procedure. Both inkjet printing and LAB possess the ability to achieve precise cell positioning, as demonstrated by their capability to accurately place individual cells within droplets [24]. However, inkjet printing faces limitations in generating complex three-dimensional architectures, while LAB relies on positioning bioink onto pre-existing scaffolds and incurs high costs. On the other hand, extrusion bioprinting offers rapid fabrication times for large-scale 3D structures but presents challenges in ensuring optimal cell survival [24]. Therefore, combining either inkjet bioprinting or LAB with extrusion printing presents an advantageous approach for creating scaffolds that exhibit both physiologically relevant proportions and support viable cells [25].

A more prospective area of research is centered around 4D bioprinting. Four-dimensional bioprinting is essentially the quality of 3D bioprinted structures to dynamically alter their shape and functionality when exposed to external stimuli [26]. Numerous research studies have been published focusing on the development and utilization of 4D bioprinting technology in various fields, including neural engineering, cardiac conduit construction, bone tissue engineering, and vascular architecture [16]. The implementation of 4D bioprinting enables the fabrication of intricate structures with exceptional precision, surpassing the limitations of traditional 3D bioprinting. Moreover, 4D bioprinting exhibits the potential to generate nano- and microstructures that can respond to diverse stimuli, including light, temperature, humidity, electric fields, and magnetic fields. Looking ahead, it has the capability to revolutionize the field of biomedical science, particularly in specific domains such as tissue engineering and regenerative medicine [16].

## Conclusions

Bioprinting, a cutting-edge approach combining biology, engineering, and materials science, holds immense promise in addressing the scarcity of organs for transplantation. This innovative technology allows the precise fabrication of personalized biological structures by replicating human tissues and organs. Bioprinting offers a solution to the overcoming limitations of traditional tissue engineering methods.

The process consists of three main stages: pre-bioprinting, bioprinting, and post-bioprinting. Bioink, the primary substance used, is created through cell selection and cultivation and typically comprises living cells, growth-promoting chemicals, and a supporting structure like a hydrogel. Bioprinting can be achieved using various methods, including robotic dispensing/extrusion, inkjet, and laser-based techniques. While bioprinting offers significant advantages, challenges remain. Issues such as cell and bioink sourcing, integration of vascular and neuronal networks, biocompatibility, disease transmission risk, and regulatory considerations must be addressed. Despite these challenges, ongoing research and technological advancements in bioprinting have the potential to revolutionize disease modeling, regenerative medicine, and tissue engineering.
